# Geographical Variation in Medication Prescriptions: A Multiregional Drug-Utilization Study

**DOI:** 10.3389/fphar.2020.00418

**Published:** 2020-05-05

**Authors:** Veronica Russo, Valentina Orlando, Valeria Marina Monetti, Federica Galimberti, Manuela Casula, Elena Olmastroni, Elena Tragni, Enrica Menditto, EDU.RE.DRUG Group, Alberico L. Catapanoa

**Affiliations:** ^1^Department of Pharmacy, University of Naples Federico II, Naples, Italy; ^2^CIRFF-Center of Pharmacoeconomics and Drug Utilization Research, University of Naples Federico II, Naples, Italy; ^3^Centro Interuniversitario di Epidemiologia e Farmacologia Preventiva, Dipartimento di Scienze Farmacologiche e Biomolecolari, Università degli Studi di Milano, Milan, Italy; ^4^MultiMedica (IRCCS), Milan, Italy

**Keywords:** drug-utilization, geographical difference, prevalence, drug use, general practitioner, pharmacy claims

## Abstract

**Background:**

Studies have emphasized the importance of geographical factors and general practitioner (GP) characteristics in influencing drug prescriptions.

**Objectives:**

To: (i) ascertain the prevalence rate (PR) of use of drugs in six therapeutic categories used for chronic conditions; (ii) assess how geographical characteristics and GP characteristics may influence drug prescribing.

**Methods:**

This study is part of the EDU.RE.DRUG Project, a national collaborative project founded by Italian Medicine Agency (AIFA). Cross-sectional analyses were undertaken employing the pharmacy-claim databases of four local health units (LHUs) located in two Italian regions: Lombardy and Campania. Six drug categories were evaluated: proton-pump inhibitors; antibiotics; respiratory-system drugs; statins; agents acting on the renin−angiotensin system; psychoanaleptic drugs. The PR was estimated according to drug categories at the LHU level. A linear multivariate regression analysis was undertaken to evaluate the association between the PR and geographical area, age and sex of GPs, number of patients, and percentage of patients aged >65 per GP.

**Results:**

LHUs in Campania showed a PR that was significantly higher than that in Lombardy. Antibiotics showed the highest PR in all the LHUs assessed, ranging from 32.5% in Lecco (Lombardy) to 59.7% in Naples-2 (Campania). Multivariate linear regression analysis confirmed the association of the PR with geographical area for all drug categories. Being located in Campania increased the possibility of receiving a drug prescription from the categories considered, with estimates more marked for antibiotics, proton-pump-inhibitors, and respiratory-system drugs.

**Conclusions:**

This study provides information about the PR of medications used for treating common and costly conditions in Italy and highlighted a significant geographical variation. These insights could help to develop area-specific strategies to optimize prescribing behavior.

## Introduction

Over the last century, advances in medical therapeutics have contributed to improve global health and to increase life expectancy. However, growing evidence suggests that therapeutic decisions are often potentially inappropriate, possibly resulting in negative outcomes, such as adverse drug events, hospitalization, and increased healthcare resource utilization (Arnold, 1999).

Appropriate prescription of medications is one of the most important components of healthcare. It reflects the accuracy of the diagnosis, adherence to evidence-based guidelines, and susceptibility to drug-marketing and regulatory factors. It is particularly challenging in older patients, mainly due to age-related changes in pharmacokinetics and pharmacodynamics, high numbers of concurrent medications, functional status, and burden of co-morbid illness (Mort and Aparasu, 2002).

A lot of researches have tried to analyze and understand the factors which influence physician prescribing decisions and practice. Among the major determinants of drug prescription, studies suggested the role of geographical differences. Scholars have shown that drug use varies across regions in Europe and the USA by more than would be expected based on population age and health status alone (Couto et al., 2014; Nolte and Corbett, 2015). Such variations may be dependent upon differences in the prescribing habits of general practitioners (GPs) and socioeconomic status of patients (Odubanjo et al., 2004). Furthermore, variations in prescription patterns among different regions and between areas within the same region in Italy have been documented (Russo et al., 2018; Orlando et al., 2020). For instance, the Italian National Observatory on Drug Prescription (OsMed) revealed that, in 2016, the overall prescription for all reimbursed drugs, expressed in defined daily doses (DDDs) per 1,000 inhabitants per day, was 900.7 in northern Italy and 1,048.8 in southern Italy (Rapporto OsMed, 2018). Such geographical variations found further confirmation in the work of Piovani et al. (Piovani et al., 2012) conducted in a pediatric population, where a strong inverse correlation between prescription patterns and latitude was observed.

Despite an increasing attention to variations in use of prescribed drugs, little is known about the drivers of variations in the prescribing patterns of GPs. Very few studies attempted to quantify the geographical variation in drug prescriptions for chronic conditions among adults and the elderly in Italian regions (Orzella et al., 2010; Franchi et al., 2013).

Recently, the Italian Medicine Agency (AIFA) funded the EDU.RE.DRUG Project (Effectiveness of informative and/or educational interventions aimed at improving the appropriate use of drugs designed for general practitioners and their patients). The EDU.RE.DRUG Project aims to evaluate the appropriateness of drug prescription in people aged ≥40 years living in Lombardy or Campania.

The present analysis is part of this national collaborative project. We wished to: (i) describe the prescription pattern for medications belonging to six therapeutic categories used for chronic conditions; (ii) assess how geographical factors and GP characteristics may influence prescription patterns.

## Materials and Methods

### Study Design

This was a retrospective drug-utilization study based on use of administrative health-related databases. The study was carried out according to the STROBE (Strengthening the Reporting of Observational Studies in Epidemiology) guidelines (STROBE).

### Study Setting and Population

Italian National Health Service (INHS) provides all citizens and legal foreign residents with economic coverage of drugs with documented clinical efficacy and which are used for treating serious and chronic diseases. The amount of public money to be spent on healthcare is established annually by the central government. The money is assigned to regions to provide the essential levels of care (LEA), which must be assured homogenously to citizens throughout the country. Each region allocates the funds to its local health units (LHUs) mainly on an age-adjusted capitation basis. Assigned funds are used by LHUs for the direct provision of inpatient and outpatient care, for GP remuneration, and for the cost reimbursement of healthcare services afforded by independent and university hospitals and/or accredited private providers (Cicchetti and Gasbarrini, 2016).

The study was conducted in the primary care setting, involving selected LHUs of two Italian regions: Lombardy and Campania. Lombardy is one of the largest Italian regions, situated in the north of the country, with a population of over 10,019,000 inhabitants. Campania is situated in the south of the country and had a population of ~5,839,000 inhabitants on 1 January 2017 (Demo-Gedemo).

The LHUs involved in the study were: Naples-1 and Naples-2 in Campania, and Bergamo and Lecco in Lombardy, with an overall population of ~3.4 million inhabitants. Patients aged ≥40 years, receiving at least one prescription of the study drugs, were included in our study.

### Sources and Collection of Data

The study data were obtained from administrative databases containing information on all beneficiaries of INHS in the LHUs involved. These databases are set-up and updated constantly by regional or local health authorities. Demographic databases contain anonymized data on residents (birth date and sex), and on prescribers (GPs) (birth date and sex) of each LHU. The pharmacy databases contain data on drug prescriptions dispensed by local pharmacies and reimbursed by INHS, including: patient's anonymous unique code; prescriber's (GP) anonymous unique code; prescription date; dispensation date; Anatomical Therapeutic Chemical (ATC) classification; marketing authorization code (AIC); number of DDDs; number of boxes; cost for INHS (WHOCC).

Pharmacy databases were linked to demographic databases by deterministic record-linkage procedures through the unique and anonymous personal identification codes. Such codes were created by a database manager, uninvolved in the data analysis, in full preservation of individuals' privacy.

Drug-utilization data obtained from these databases have been validated previously and used in drug-utilization studies (Iolascon et al., 2013; Cammarota et al., 2014; Casula et al., 2014; Iolascon et al., 2016; Putignano et al., 2017; Menditto et al., 2018).

### Drug Categories

We analyzed prescriptions of drugs belonging to six therapeutic categories, pre-selected based on the higher prevalence in terms of gross public expenditure and consumption.

The six therapeutic categories were: proton pump inhibitors (PPIs) [A02BC]; antibiotics [J01]; respiratory-system drugs (RSDs) [R03]; 3-hydroxy-3-methyl-glutaryl-CoA reductase inhibitors [C10AA]; agents acting on the renin–angiotensin system (C09, including angiotensin-converting enzyme inhibitors [C09AA] and angiotensin-II antagonists [C09CA]) and psychoanaleptic drugs (N06, including selective serotonin reuptake inhibitors [N06AB] and other antidepressants [N06AX]).

In Italy, drugs for the treatment of chronic conditions are fully covered by INHS and, therefore, traceable through administrative databases.

### Study Outcomes

The year considered for this analysis was 2016. Medication use for the identified therapeutic categories among adults (≥40 years) was estimated as prevalence rate (PR), calculated for each GP as the proportion of patients who received at least one prescription of the selected drugs per 100 GP patients of the same age range in 2016 The mean PR at the LHU level was adjusted by age using a direct standardization method whereby the standard population (also known as the “reference population”) was the Italian population as extrapolated from Italian Statistical Agency (ISTAT) data on 1 January 2017.

### Statistical Analyses

Continuous variables (number of patients per GP and age of GP) are expressed as median and interquartile-range deviation, as the Shapiro-Wilk test showed that they did not have a normal distribution. Categorical data are given as percentage.

PR at the LHU level was expressed as mean GP's PR and 95% confidential interval. ANOVA was used to compare the distributions of PRs by LHUs.

A multivariate linear regression analysis was undertaken for each selected therapeutic category. The PR was the dependent variable, whereas the geographical area, number of patients per GP, age of GP, sex of GP, and percentage of patients aged >65 years per GP were inserted as independent variables.

Data management was undertaken with Microsoft SQL server (version 2018). Statistical analyses were carried out with SPSS v17.1 (IBM, Armonk, NY, USA). P < 0.05 was considered significant.

## Results

**Figure 1:**
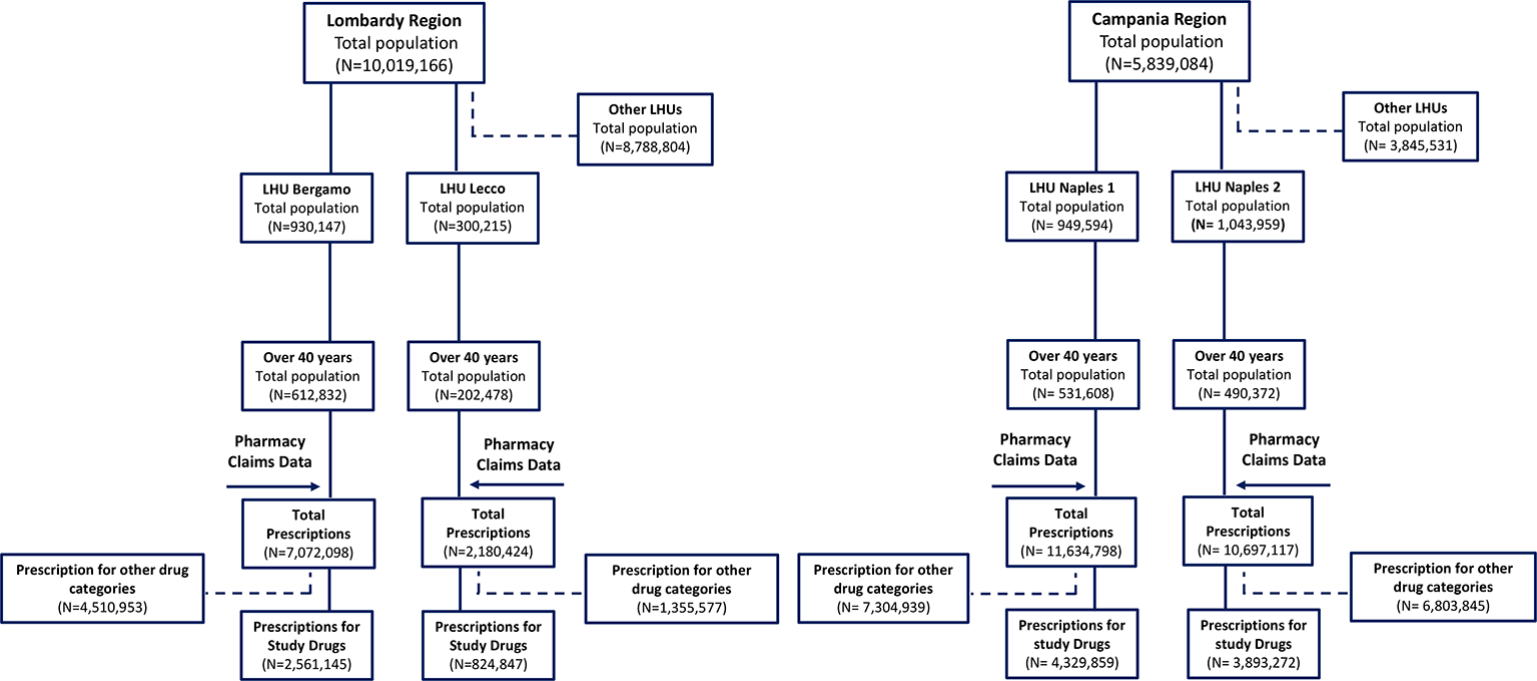
Flowchart of data selection.

In 2016, for an overall population of 15,858,250 (5,839,084 in Campania and 10,019,166 in Lombardy) GPs dispensed 31,584,437 prescriptions (22,331,915 prescriptions for Campania and 9,252,522 prescriptions for Lombardy). Prescriptions for ATC-selected categories (n = 11,609,123) accounted for 36.7% of the total drugs prescribed. **Figure 1** shows the regional databases available and data selection employed.

**Table 1** shows the main characteristics of GPs of the four LHUs. The number of GPs ranged from 205 in Lecco to 777 in Naples-2.

**Table 1:** GP characteristics at the LHU level.

	Northern Italy		Southern Italy		Total LHUs
	Bergamo LHU	Lecco LHU	Naples-1 LHU	Naples-2 LHU	(2, 956, 990 patients aged ≥ 40 years)
	(930, 124 patients aged ≥ 40 years)	(300, 215 patients aged ≥ 40 years)	(832, 780 patients aged ≥ 40 years)	(893, 871 patients aged ≥ 40 years)	
GPs (N)	660	205	718	777	2,360
Patients per GP, median (IQR)	1,519 (257)	1,553 (233)	1,267 (528)	1,305 (669)	1,401.50 (470)
Patients aged ≥ 65 years per GP (%)	23.0	25.2	22.8	15.0	21.0
Age of GP, median (IQR)	59 (9)	60 (8)	62 (6)	60 (6)	61 (7)
Sex of GP					
M (%)	67.1	71.2	78.7	81.9	75.8
F (%)	32.9	28.8	21.3	18.1	24.2

Most GPs were men (75.8%) and the median age was ~61 years.

The percentage of patients aged ≥65 years per GP was 21.0%, with geographical variability (ranging from 15.0% for Naples-2 to 25.2% for Lecco; p < 0.001). A regional difference regarding the median number of patients for each GP (~1500 in northern Italy *versus* ~1300 in southern Italy) was noted.

### PR at the LHU Level

**Table 2** (and **Supplementary Table S1**) shows PR for each selected drug category stratified by LHU (and for age class: 40–64 and >=65 years old). A significant difference in the PR among LHUs was documented: in particular, LHUs located in the south of Italy showed a higher PR compared with LHUs located in the North of Italy for all drug categories (*p* < 0.001).

**Table 2:** Prevalence rate of medication use by ATC group at the LHU level.

	Northern Italy				Southern Italy				p*
	Bergamo LHU		Lecco LHU		Naples-1 LHU		Naples-2 LHU		
	Standardized prevalence %	95% CI	Standardized prevalence %	95% CI	Standardized prevalence %	95% CI	Standardized prevalence %	95% CI	
A02BC	24.1	(23.9; 24.2)	21.9	(21.7; 22.1)	40.0	(39.9; 40.2)	41.3	(41.1; 41.5)	<0.001
C09	16.4	(16.3; 16.5)	19.8	(19.6; 20.0)	21.9	(21.8; 22.0)	22.2	(22.1; 22.4)	<0.001
C10AA	15.3	(15.2; 15.4)	13.9	(13.7; 14.0)	19.3	(19.2; 19.4)	22.6	(22.4; 22.7)	<0.001
J01	35.3	(35.1; 35.4)	32.5	(32.3; 32.8)	53.9	(53.7; 54.1)	59.7	(59.4; 59.9)	<0.001
N06	8.7	(8.6; 8.8)	7.0	(6.9; 7.1)	8.3	(8.3; 8.4)	8.6	(8.5; 8.7)	<0.001
R03	10.3	(10.2; 10.4)	9.4	(9.3; 9.6)	20.4	(20.2; 20.5)	21.8	(21.6; 21.9)	<0.001

A02BC: Proton pump inhibitors; C10AA: HMG CoA reductase inhibitors; J01: antibiotics; RO3: respiratory-system drugs;

C09: ACE inhibitors (C09AA) + angiotensin-II antagonists (C09CA); N06: selective serotonin reuptake inhibitors (N06AB) + another antidepressants (N06AX).

*ANOVA.

Among the drug categories evaluated, antibiotics showed the higher PR, ranging from 32.5% in Lecco to 59.7% in Naples-2. Psychoanaleptic drugs showed the lowest PRs, with the smallest variations among LHUs (8.7% in Bergamo, 7.0% in Lecco, 8.3% Naples-1, and 8.6% Naples-2). PR for PPIs (A02BC) ranged from 21.9% in Lecco to 41.3% in Naples-2.

### Multivariate Analyses

The regression model (**Table 3** and **Supplementary Tables S2** and **S3**) showed an association of the PR with geographical area for all drug categories, with lower PRs in Northern Italy compared to Southern Italy. The coefficients were notable for: (i) antibiotics; (ii) PPIs; (iii) RSDs. Indeed, the average PR for antibiotics was ~21% lower in northern Italy than in southern Italy (B: −21.80; 95% confidence interval [CI]: −22.65 to −20.97; p < 0.001). For PPIs, northern Italy showed a lower mean PR compared with that in southern Italy (B: −17.33; 95% CI: −18.08 to −16.57; p < 0.001). Similar findings were observed for RSDs (B: −10.76; 95% CI: −11.30 to −10.22; p < 0.001).

**Table 3:** Multivariate linear regression (95% CI): prevalence rate (%) for general practitioners (n = 2,360).

Characteristic	A02BC (R^2^ = 0.509)			C09 (R^2^ = 0.305)			C10AA (R^2^ = 0.290)			J01 (R^2^ = 0.616)			N06 (R^2^ = 0.068)			R03 (R^2^ = 0.471)		
	B	95% CI	*p*	B	95% CI	*p*	B	95% CI	*P*	B	95% CI	*p*	B	95% CI	*p*	B	95% CI	*p*
**Geographical area**																		
**Southern Italy**	Reference			Reference			Reference			Reference			Reference			Reference		
**Northern Italy**	− 17.33	(−18.08; −16.57)	<0.001	−5.74	(−6.13;−5.34)	<0.001	−623	(−6.65; −5.82)	<0.001	−21.80	(−22.65; −20.97)	<0.001	−0.71	(−0.97; −0.45)	0.001	−10.76	(−11.30;−10.22)	<0.001
Patients per GP*	0.61	(0.47; 0.75)	<0.001	0.24	(0.16; 0.31)	<0.001	0.40	(0.32; 0.47)	<0.001	0.91	(0.76; 1.07)	<0.001	0.11	(0.06; 0.16)	<0.001	0.35	(0.25; 0.45)	<0.001
**Age of GP**	−0.04	(−0.10; 0.15)	0.152	−0.05	(−0.08; 0.02)	0.001	−0.04	(-0.07; −0.01)	0.019	−0.12	(−0.18; −0.05)	<0.001	−0.06	(−0.07; −0.04)	<0.001	−0.08	(−0.12; −0.04)	<0.001
**Sex of GP**																		
**M**	Reference			Reference			Reference			Reference			Reference			Reference		
**F**	−0.22	(−0.95; 0.51)	0.554	0.44	(0.06; 0.82)	0.022	−0.20	(−0.60; 0.19)	0.313	−0.955	(−1.77; −0.14)	0.021	0.16	(−0.09; 0.41)	0.223	0.16	(−0.36; 0.68)	0.550
**% Patients ≥ 65 years per GP**	0.10	(0.05; 0.15)	<0.001	0.26	(0.23; 0.29)	<0.001	0.13	(0.10; 0.16)	<0.001	−0.371	(−0.43; −0.31)	<0.001	0.09	(0.07; 0.10)	<0.001	−0.11	(−0.15; −0.07)	<0.001

*Patients per GP has been multiplied by 100.

With regard to the number of patients per GP, we observed a significant positive association for all selected drugs. Similar results were observed with the percentage of patients aged >65 years per GP, with the exception of a negative association for antibiotics (B: −0.37; 95% CI: −0.43; −0.31; p < 0.001) and RSDs (B: −0.11; 95% CI: −0.15 to −0.07; p < 0.001). We also observed a trend in decreasing PRs with increasing age of the GP.

The sex of GPs was significantly associated with the PR for drugs based on the renin–angiotensin system (B: 0.44; 95% CI: 0.06 to 0.82; p = 0.022) and for antibiotic treatments (B: −0.96; 95% CI: −1.77 to −0.14; p = 0.021): female GPs had a higher C09 mean PR compared with male GPs, while low average of antibiotic PR.

## Discussion

The prescribing of medicines is a complex process that goes on in every healthcare setting, and whose principles are based on the doctor's choice of the right drug for the right patient (Abrahams, 2008).

Several studies suggested that some factors may have a role in influencing the physicians' prescribing behavior. Such factors for instance include, the age and sex of the physician or the patient, or the socio-economic characteristics of the practicing area (Bradley, 1992).

In our study, we highlighted that some characteristics of GPs and geography might affect the prescribing of drugs in terms of the PR.

In particular, a higher PR in southern Italy than in northern Italy was observed. The greatest difference in the PR between regions was observed for antibiotics, PPIs, and RSDs.

This finding is in accordance with observations from other studies conducted in a similar setting (Piovani et al., 2012; Piovani et al., 2014; Di Martino et al., 2017). Piovani et al. in 2012 showed a mean antibiotic PR of 46.5% in the North of Italy, while Southern Regions showed a mean PR of 61.1% (Piovani et al., 2012). This geographical difference was observed again in a subsequent study by the same authors, indicating that the PR of antibiotics and RSDs in Southern Regions was higher (up to 57.5% and 27.0%, respectively) than in the rest of Italy. The authors pointed out the role of socioeconomic and sociodemographic factors in explaining a higher PR in south Italy (Piovani et al., 2014). Furthermore, studies analyzing the PR for antibiotics in Campania showed results consistent with our data, and indicated that the PR was also influenced by per capita income (Favato et al., 2007; Orzella et al., 2010; Di Martino et al., 2017).

Notably, the selected drug categories include medications used to treat common and costly conditions, and the observed geographical differences may reflect differences in socio-demographic indicators between the two Italian Regions. Indeed, the differences observed between the two regions in terms of prescription of drugs for chronic diseases may also be due to differences in the socioeconomic context of the two regions. According to a recent report from ISTAT, southern regions have a lower income and socioeconomic level than those of northern Italy. Indeed, the gross domestic product (GDP) is ~€18,216 in Campania compared with ~€36,807 in Lombardy Rapporto Oasi-Osservatorio sulle Aziende e sul Sistema sanitario Italiano (2017). **Supplementary Table S4** shows demographic and socioeconomic characteristics by geographical area. The Lombardy region, with 22.2% of patients aged ≥65 years, had an older population than the Campania region (18.2%). Private health expenditure per household showed quite high geographical variability (ranging from €752 in the north to €303 in the south). Similar findings were observed for public-health expenditure per capita (ranging from €3,452.4 in Lombardy to €1,479.6 in Campania). There is evidence that a lower income and lower level of education are associated with greater use of health services reimbursed by INHS (Grosso et al., 2012). Although we have no data to confirm that hypothesis in our study, we can assume that a higher GDP and higher level of education in Lombardy can be associated with a lower PR of reimbursed drugs for chronic conditions (Bianchi et al., 2011).

Furthermore, geographical differences in the prescribing patterns between different areas of the same country may also have several causes beyond socioeconomic differences. In Italy, many health policies are designed and implemented at Regional level. Among these, there may be indications orienting the prescribing practice, essentially with the aim of cost containment (for example, towards the choice of drugs with comparable efficacy but lower costs) or of risk minimization (for example, towards the choice of drugs associated with a better tolerability profile of with less reporting of medication errors or adverse events) (Russo et al., 2018; Orlando et al., 2020).

Other aspects of a GP's practice appear to have an impact on the prevalence rates. In our study, a high number of patients per GP was associated with a greater likelihood of receiving prescriptions. This relationship may be because a high number of listed patients may imply a greater diversity of illnesses and, consequently, a greater diversity of therapeutic needs (Tamblyn et al., 2003; Scala et al., 2016). Moreover, GPs with a high percentage of people aged >65 years showed greater PRs. This finding, consistent with data from other studies, could be explained by the higher number of comorbidities usually affecting elderly patients (Tamblyn et al., 2003). Interestingly, the stratification of the regression by Region revealed that the positive association between older age of GP's patients and PR was more marked in Lombardy than in Campania (**Supplementary Table S2** and **S3**).

Several associations found in our analysis were already been observed by other researchers. Orzella et al. showed that younger GPs were more likely to prescribe medications and suggested that the number of years in practice could be a proxy of prescribing behavior (Orzella et al., 2010). The sex of the GP did not seem to have an influence on the PRs. However, consensus on the influence of this factor on the prescribing attitude is lacking. Some studies showed that female GPs are more likely to write prescriptions (Morrison et al., 2009; Orzella et al., 2010), whereas in other studies male GPs had higher rates of drug use than females (Tamblyn et al., 2003; Haastrup et al., 2016).

Our study had two main limitations. First, pharmacy-claim data do not contain information about over-the-counter (OTC) medications and out-of-pocket expenditure, which could imply underestimation. Second, a dispensed prescription does not ensure that the medication was consumed by the patient. This, in turn, implies that the PR may be an overestimate, as some individuals filled out their prescriptions but did not take the drug. Nevertheless, these two limitations are common in drug-utilization studies carried out with administrative data.

Importantly, our study was not designed nor aimed to assess the appropriateness of drug prescription. Therefore, the quantification of use of the selected drug categories does not imply a qualitative judgment *per se*, as our data did not allow us to evaluate if these prescriptions were appropriate. The purpose of our analysis was to provide a picture of the PR for prescription of major drug groups with respect to the geographical area in which they were prescribed and the characteristics of prescribers. Further research is required to achieve consistent assessment of the level of potential prescribing inappropriateness among GPs. These actions have been envisaged as the next steps in the EDU.RE.DRUG project. The present work represents the first step of this research project.

On the other hand, as a main strength of our study, we used a population-based database, covering a defined and stable population, which corresponded to ~26% of the Italian population (Cicchetti and Gasbarrini, 2016). The age distribution and sex distribution of the population was similar to the total Italian population. Therefore, we believe that this study provides a reliable representation of Italy, with an up-to-date overview of drug use and an evaluation of the relationship between geographical areas, GP characteristics, and the PR of drug prescription in a real-life context.

Although it is important to be aware of the limitations of cross-sectional studies and their usefulness in the formulation of future interventions (Illario et al., 2015; Illario et al., 2016), our data could be very useful in planning interventions aimed at improving the practice of drug prescribing. Successful elements from activities in other countries should also be implemented.

## Conclusion

Geographical variations and GP characteristics are associated with prescription of medications for treating common and costly conditions in Italy. This study is the first step towards the characterization of these differences, and future work is needed to deepen and understand the reasons behind these geographical differences.

## Data Availability Statement

The datasets for this manuscript are not publicly available because the data set was only accessed and analyzed by the authors who are affiliates to the Institutions involved in the EDU.RE.DRUG project. Authors who are not affiliates received the results from the analysis of the data for discussion. Requests to access these datasets should be directed to the corresponding author.

## Ethics Statement

Ethical approval for carrying out the present study was granted by the Ethics Committee of the University of Milan (Milan, Italy) in June 2017 (code 15/17) and of the LHU Naples-1 in October 2017 (code 2017/0091873). Procedures aimed at protecting personal data were implemented to safeguard privacy and to prevent the identification of individual data (according to Italian law D.Lgs. n. 196/2003). Anonymized regional administrative data can be used without specific written informed consent if patient information is collected for the management quality evaluation and improvement of healthcare (according to article 110 on medical and biomedical and epidemiological research, Legislation Decree 101/2018).

## Collaborative Authors

The list of collaborators of the group and their affiliation are the following:

The EDU.RE.DRUG Group
Alberico L. Catapanoa^2^,
Elena Tragni^1^, Enrica Menditto^3^, Giovanni Corrao^4^,
Manuela Casula^1^^,^ ^2^ Federica Galimberti^1^, Elena Olmastroni^1^,
Veronica Russo^3^, Valentina Orlando^3^, Sara Mucherino^3^,
Lorenza Scotti^4^, Antonella Zambon^4^,
Marco Gambera^5^, Rossana Piccinelli^5^, Samanta Sonzogni^5^,
Valter Valsecchi^6^, Eugenio Scopinaro^6^,
Sandro Raineri^7^, Alessia Speziali^7^,
Simona Creazzola^8^, Michele Tari^9^, Mariano Fusco^10^

## Author Contributions

VR: Writing—original draft, visualization. VO: Conceptualization, methodology, writing—review and editing. VM: Methodology, software, formal analysis. FG: Writing—review and editing. MC: Writing—review and editing. EO: Writing—review and editing. ET: Project administration, writing—review and editing. EM: Supervision, project administration, conceptualization, writing—review and editing.

## Funding

This study was supported by a research grant from the Italian Medicines Agency (FARM:12KSBT).

## Conflict of Interest

The authors declare that the research was conducted in the absence of any commercial or financial relationships that could be construed as a potential conflict of interest.

## Footnotes

1 Epidemiology and Preventive Pharmacology Service (SEFAP), Department of Pharmacological and Biomolecular Sciences, University of Milan, Milan, Italy

2 IRCCS MultiMedica Hospital, Sesto S. Giovanni, Milan, Italy

3 CIRFF, Center of Pharmacoeconomics, Federico II University of Naples, Naples, Italy

4 Department of Statistics and Quantitative Methods (DiSMeQ), University of Milano‐Bicocca, Milan, Italy

5 Bergamo Local Health Authority, Bergamo, Italy

6 Lecco Local Health Authority, Lecco, Italy

7 Mantova Local Health Authority, Mantova, Italy

8 Local Health Authority Napoli1 Centro, Naples, Italy

9 Local Health Authority Caserta, Caserta, Italy

10 Local Health Authority Napoli2 Nord, Frattamaggiore (NA), Italy

## Abbreviations

AIC, Marketing Authorization Code; AIFA, Italian Medicine Agency; ATC, Anatomical Therapeutic Chemical classification; DDD, Defined daily dose; GP, General practitioner; INHS, Italian National Health Service; ISTAT, Italian Statistical Agency; LHU, Local health unit OsMed: Italian National Observatory on Drug Prescription; OTC, Over-the-counter; PPI, Proton pump inhibitor; PR, Prevalence rate; RSD, Respiratory-system drug.
