# Supplementary material for: Geographical Variation in Medication Prescriptions: A Multiregional Drug-Utilization Study
Source: Front Pharmacol. 2020 May 5;11:418. doi: 10.3389/fphar.2020.00418 (PMC7269055; doi:10.3389/fphar.2020.00418)
Supplement: Supplementary file 1 [file Table_1.docx]

**Supplementary Table S1 Mean prevalence rate by LHUs and age group**

|  | **Northern Italy** | | **Southern Italy** | |  |
| --- | --- | --- | --- | --- | --- |
|  | **Bergamo LHU** | **Lecco LHU** | **Naples-1 LHU** | **Naples-2 LHU** | **ANOVA** |
|  | **Mean (±SD)** | **Mean (±SD)** | **Mean (±SD)** | **Mean (± SD)** | ***p*** |
| **40−64 years** | | | | | |
| **A02BC** | 14.2 (±5.1) | 13.8 (±4.5) | 27.5 (±8.6) | 28.6 (±8.5) | <0.001 |
| **C09** | 8.8 (±2.2) | 10.7 (±2.5) | 12.7 (±3.6) | 14.0 (±3.5) | <0.001 |
| **C10AA** | 6.8 (±2.5) | 6.1 (±2.3) | 9.8 (±3.5) | 12.7 (±3.6) | <0.001 |
| **J01** | 31.0 (±6.0) | 29.1 (±5.5) | 46.7 (±10.3) | 53.1 (±9.6) | <0.001 |
| **N06** | 5.7 (±1.8) | 5.1 (±1.5) | 5.6 (±2.4) | 5.9 (±2.3) | <0.001 |
| **RO3** | 8.2 (±2.7) | 7.8 (±2.5) | 16.1 (±6.0) | 17.1 (±5.8) | <0.001 |
| **≥65 years** | | | | | |
| **A02BC** | 40.4 (±9.1) | 34.8 (±7.7) | 58.9 (±10.7) | 61.2 (±10.5) | <0.001 |
| **C09** | 28.9 (±5.2) | 34.5 (±6.0) | 36.1 (±7.9) | 35.3 (±7.8) | <0.001 |
| **C10AA** | 29.2 (±6.8) | 26.5 (±5.7) | 33.9 (±7.7) | 37.4 (±7.6) | <0.001 |
| **J01** | 42.2 (±7.4) | 37.4 (±6.2) | 63.7 (±12.0) | 68.9 (±11.1) | <0.001 |
| **N06** | 13.4 (±4.0) | 9.9 (±3.2) | 12.4 (±4.8) | 12.7 (±5.3) | <0.001 |
| **RO3** | 13.8 (±4.3) | 11.9 (±3.4) | 26.8 (±8.7) | 29.4 (±9.2) | <0.001 |
