# Supplementary material for: Geographical Variation in Medication Prescriptions: A Multiregional Drug-Utilization Study
Source: Front Pharmacol. 2020 May 5;11:418. doi: 10.3389/fphar.2020.00418 (PMC7269055; doi:10.3389/fphar.2020.00418)
Supplement: Supplementary file 2 [file Table_2.docx]

**Supplementary Table S2 Multivariate linear regression for Campania (95%CI)**

| **Characteristics** | **A02BC**  **(R^2^=0.055)** | | | | **C09 (R^2^=0.064)** | | | **C10AA**  **(R^2^=0.100)** | | | **J01 (R^2^=0.034)** | | | **N06 (R^2^=0.028)** | | | **R03 (R^2^=0.014)** | | |
| --- | --- | --- | --- | --- | --- | --- | --- | --- | --- | --- | --- | --- | --- | --- | --- | --- | --- | --- | --- |
|  | **B** | | **95%CI** | ***p*** | **B** | **95%CI** | ***p*** | **B** | **95%CI** | ***p*** | **B** | **95%CI** | ***p*** | **B** | **95%CI** | ***p*** | **B** | **CI** | ***p*** |
| **Patients per GP*** | | 0.8 | (0.6; 0.9) | <0.001 | 0.5 | (0.4; 0.5) | <0.001 | 0.6 | (0.5; 0.7) | <0.001 | 0.6 | (0.4; 0.8) | <0.001 | 0.2 | (0.1; 0.2) | <0.001 | 0.3 | (0.2; 0.4) | <0.001 |
| **Age of GP** | 0.01 | | (−0.08; −0.10) | 0.751 | 0.03 | (−0.02; 0.08) | 0.193 | 0.01 | (−0.04; 0.06) | 0.730 | -0.23 | (−0.34; −0.13) | <0.001 | −0.02 | (−0.05; 0.00) | 0.105 | −0.08 | (−0.15; −0.01) | 0.027 |
| **Sex of GP** |  | | |  |  | |  |  | |  |  | |  |  | |  |  | |  |
| **M** | Reference | | | Reference | | | Reference | | | Reference | | | Reference | | |  | Reference | |  |
| **F** | −0.14 | | (−1.21; 0.92) | 0.793 | 0.46 | (−0.11; 1.04) | 0.114 | −0.09 | (−0.65; 0.46) | 0.743 | −1.06 | (−2.33; 0.21) | 0.102 | 0.05 | (−0.30; 0.41) | 0.772 | 0.07 | (−0.76; 0.89) | 0.869 |

*Patients per GP has been multiplied by 100
