# Supplementary material for: Geographical Variation in Medication Prescriptions: A Multiregional Drug-Utilization Study
Source: Front Pharmacol. 2020 May 5;11:418. doi: 10.3389/fphar.2020.00418 (PMC7269055; doi:10.3389/fphar.2020.00418)
Supplement: Supplementary file 3 [file Table_3.docx]

**Supplementary Table S3 Multivariate linear regression for Lombardy (95%CI)**

| **Characteristic** | **A02BC (R^2^=0.020)** | | | **C09**  **(R^2^=0.070)** | | | **C10AA (R^2^=0.026)** | | | **J01 (R^2^=0.023)** | | | **N06 (R^2^=0.046)** | | | **R03 (R^2^=0.055)** | | |
| --- | --- | --- | --- | --- | --- | --- | --- | --- | --- | --- | --- | --- | --- | --- | --- | --- | --- | --- |
|  | **B** | **95%CI** | ***p*** | **B** | **95%CI** | ***p*** | **B** | **95%CI** | ***p*** | **B** | **95%CI** | ***p*** | **B** | **95%CI** | ***p*** | **B** | **95%CI** | ***p*** |
| **Patients per GP*** | 0.45 | (0.2; 0.7) | <0.001 | 0.5 | (0.4; 0.7) | <0.001 | 0.3 | (0.2; 0.5) | <0.001 | 0.004 | (0.2; 0.7) | <0.001 | 0.2 | (0.1; 0.3) | <0.001 | 0.1 | (0.0; 0.2) | 0.011 |
| **Age of GP** | −0.05 | (−0.11; 0.01) | 0.123 | −0.06 | (−0.10; 0.02) | 0.002 | −0.03 | (−0.07; 0.00) | 0.084 | -0.09 | (−0.16; −0.03) | 0.003 | -0.06 | (-0.09; -0.04) | <0.001 | −0.09 | (−0.12; −0.06) | <0.001 |
| **Sex of GP** |  | |  |  | |  |  | |  |  | |  |  | |  |  | |  |
| **M** | Reference | | Reference | | | Reference | | |  | Reference | |  | Reference | | | Reference | |  |
| **F** | −0.40 | (−1.29; 0.49) | 0.373 | 0.21 | (−0.32; 0.75) | 0.436 | −0.45 | (−1.01; 0.12) | 0.124 | −0.53 | (−1.41; 0.36) | 0.242 | 0.22 | (−0.13; 0.57) | 0.216 | 0.34 | (−0.09; 0.77) | 0.118 |

*Patients per GP had been multiplied by 100
