# Supplementary material for: Geographical Variation in Medication Prescriptions: A Multiregional Drug-Utilization Study
Source: Front Pharmacol. 2020 May 5;11:418. doi: 10.3389/fphar.2020.00418 (PMC7269055; doi:10.3389/fphar.2020.00418)
Supplement: Supplementary file 4 [file Table_4.docx]

**Supplementary Table S4 Demographic and socioeconomic characteristics, 2016**

|  | **Lombardy** | **Campania** | **Italy** |
| --- | --- | --- | --- |
| **Demographic characteristics** | | | |
| Population size (N)^[[1]](#footnote-1)^ | 10,019,166 | 5,839,166 | 60,589,445 |
| Patients aged ≥65 years (%)^1^ | 22.2 | 18.2 | 22.3 |
| **Socio-economic characteristics** | | | |
| GPD per capita (Euro)^1^ | 36,807.08 | 18,216.76 | 27,718.82 |
| Poverty rate (%)^1^ | 5.5 | 24.4 | 12.3 |
| Private health expenditure per household (Euro)^[[2]](#footnote-2)^ | 752 | 303 | 560 |
| Public health expenditure (per capita) (Euro)^[[3]](#footnote-3)^ | 3,452.4 | 1,479.6 | 2,466.0 |

1. Available from: <https://www.istat.it/>. Accessed November, 2018. [↑](#footnote-ref-1)
2. Rapporto Oasi 2017. Available from: <https://www.pensionaticisllombardia.it/public/pdf/pdf_2426_rapporto-oasi-2017.pdf>. Accessed November, 2018. [34] [↑](#footnote-ref-2)
3. Rapporto OsMed [webpage on the Internet]. 2017. Available from: http:// [www.aifa.gov.it/content/luso-dei-farmaci-italia-rapporto-osmed-201](http://www.aifa.gov.it/content/luso-dei-farmaci-italia-rapporto-osmed-2016)7. Accessed November, 2018. Italian [with English abstract]. [8] [↑](#footnote-ref-3)
